# Antibiotic Resistance in Microbes from Street Fruit Drinks and Hygiene Behavior of the Vendors in Delhi, India

**DOI:** 10.3390/ijerph17134829

**Published:** 2020-07-04

**Authors:** Neha Sharma, Kamalpreet Singh, Devinder Toor, Somnath S. Pai, Rishika Chakraborty, Khalid M. Khan

**Affiliations:** 1 Amity Institute of Virology and Immunology, Amity University Uttar Pradesh, Noida 301313, India; nehasharma.v10@gmail.com (N.S.); kamal9212114513@gmail.com (K.S.); dtoor@amity.edu (D.T.); sspai@amity.edu (S.S.P.); 2 Department of Environmental and Occupational Health, School of Public Health, Indiana University-Bloomington, Bloomington, IN 47405, USA; rchakra@iu.edu; 3 Department of Population Health, College of Health Sciences, Sam Houston State University, Huntsville, TX 77340, USA

**Keywords:** fruit juice, *E. coli*, *Salmonella*, *Vibrio*, antimicrobial resistance, hygiene

## Abstract

Microbial contamination of fruit juices has caused major outbreaks, leading to significant morbidity and mortality in developing countries. The inept hygiene and safety practices followed by the juice vendors are the leading risk factors of the microbial contamination of juices. In this pilot study, the five most crowded markets in urban Delhi, including Kamla Nagar, University of Delhi (north campus), Tilak Nagar, Chandni Chowk, and Rohini, were selected for a questionnaire survey on the fruit juice vendors and the sampling of water used for juice preparation as well as sugarcane, orange, and mix fruit juices collected from these markets for the enumeration of total bacterial count (TBC), *Escherichia coli, Salmonella*, and *Vibrio*. Antibiotic susceptibility tests were performed using ampicillin, cefotaxime, chloramphenicol, ciprofloxacin, and imipenem. The results indicated that the majority of the vendors were not following hygiene and safety practices when compared with the recommended standard safety practices. The use of municipal water by 95% of vendors with high TBC counts might have been the major source of microbial contamination in all types of fruit juices. *E. coli* and *Salmonella* contaminations were high in sugarcane (2 × 10^5^ colony forming units (CFU)/mL) and mix fruit (2.2 × 10^5^ CFU/mL) juice samples, respectively. On the other hand, *Vibrio* was found to be absent in almost all juice samples except for orange juice. All strains were found to be susceptible to chloramphenicol, but resistant to ampicillin and cefotaxime. Only a few strains were resistant to ciprofloxacin, and only *E. coli* strains were resistant to imipenem. Taken together, the overall microbiological standards of fruit juices served by street vendors were not within the acceptable limits, perhaps due to the poor quality of water used to prepare juices and poor hygiene and safety practices followed by the vendors. More importantly, the isolated microbes demonstrated resistance to ampicillin and cefotaxime, which may have pressing public health implications. Post hoc power analyses identified the minimum sample size required for 80% power.

## 1. Introduction

The consumption of street foods and drinks has increased remarkably in most low- and medium-income countries (LMIC), because of the low startup cost for the vendors and the affordable price for the people with low socio-economic status. About 2.5 billion people, mostly from the LMICs, consume street foods every day [1]. Contaminated fruit and vegetable juices have been reported to be associated with infectious disease outbreaks, producing high morbidity and mortality across the world [2,3,4,5,6]. The World Health Organization (WHO) has reported more than 600 million cases of food-related diseases in the world, resulting in 420,000 deaths and 27,000,000 years of life lost (YLL) [7]. To encounter this important public health issue, government agencies and regulatory bodies in LMIC have been trying to impose food safety rules and regulations on street-vended foods and drinks [4,7,8].

In India, millions of street food vendors are found in the metropolitan areas, representing about 2% of the total population [8,9,10]. Although these vendors contribute significantly to the economy, they are also responsible for creating potential public health challenges via foodborne illnesses. More than 100 million cases of food-borne diseases were reported in this country in 2001, and it is predicted to reach between 150 and 177 million by 2030 [11]. Among different products sold by the food vendors in urban India, fruit juice is the most popular, especially during the long hot and humid seasons. However, lack of education among the vendors on food safety and hygiene practices create opportunities of high microbial contamination of the products, including fruit juices.

Major microorganisms commonly found in street juice include *Escherichia coli*, *Salmonella typhi*, *Pseudomonas* spp, *Staphylococcus aureus* and *Vibrio cholerae.* These pathogens are linked to typhoid fever, food poisoning, gastroenteritis, enteric fever and diarrhea, which, in many cases, become life threatening across the globe [4,12,13,14]. For instance, *Salmonella typhi* has been detected in orange juice samples in Mexico and the United States [15,16], and *Escherichia coli* and *Vibrio cholera* have been commonly found in Bangladesh and Japan [17,18]. The microbial contamination of fruit juices sold by street vendors has been reported in urban India, including the crowded places of Delhi, Uttarakhand, Punjab, Kolkata, Kerala, Hyderabad and Haryana [3,19,20]. The numbers of pathogens in the fruit juices are often reported to be above the permissible standards. More importantly, the public health challenge associated with contaminated juices has aggravated in recent decades, due to the emergence of antimicrobial resistance (AMR).

Previous studies have reported the presence of AMR bacteria in apple and orange juice samples [21,22,23,24,25,26,27]. A recent study in India found 80% of the bacterial strains isolated from orange juice samples to be multi-drug resistant [28]. Another study in the same country isolated *E. coli*, *Klebsiella* spp, *Enterobacter* spp and *Enterococci* spp. from apple and pineapple juices, which were resistant to amikacin, ampicillin, cefixime, and cefotaxime [29]. However, data are limited regarding the associations between hygiene and fruit handling practices among the street juice vendors and the microbiological profile of the juices including the AMR characteristics. In order to address this knowledge gap, we conducted a pilot study to analyze fruit juice and water samples collected from street juice vendors from the five most popular and crowded markets of Delhi. The goal of the study was to generate preliminary data on the microbiological quality and AMR properties of the pathogens commonly found in fruit juice samples. To the best of our knowledge, this is the first study to report microbial profile and AMR in fruit juice samples from urban Delhi.

## 2. Materials and Methods

### 2.1. Recruitment, Questionnaire Survey, and Sample Collection

Five crowded and popular urban street markets in Delhi—Kamla Nagar (KN), University of Delhi (DU) North Campus, Tilak Nagar (TN), Chandni Chowk (CC), and Rohini (RO)— were selected for the study. Fifty fruit juice vendors, ten vendors from each street market, were randomly selected. A face-to-face questionnaire survey was administered by the field research assistants (RAs) of the projects on the juice vendors, to collect data regarding their food handling practices and personal hygiene behavior. An additional checklist was used to directly observe and record the food preparation and safety behavior and activities of the vendors by the RAs during juice preparation. All safe and unsafe hygiene practices and behaviors of the participating vendors, collected either by face-to face interview or direct observations, are listed in Table 1. Verbal consent was obtained from all the participating vendors prior to the survey and observation.

From each location, 3 sugarcane, 3 mix fruit and 4 orange juice samples were randomly collected from the vendors. Samples were collected only from the freshly prepared juices. Additionally, a total of ten water samples were collected from two randomly selected fruit juice vendors from each of the five study locations. Samples were collected in sterile containers and immediately kept at 4 °C using an ice box. Aseptic conditions were maintained during the collection of samples, which were then transported back to the microbiology laboratory of Amity University Uttar Pradesh for further analysis. All samples were processed within 4 h of collection.

### 2.2. Microbiological Characterization

#### 2.2.1. Enumeration of Bacterial Population (Total Bacterial Count)

All the samples were diluted with 0.85% NaCl, and inoculated on nutrient agar plates under sterile condition. Plates were incubated at 37 °C for 24 h. Colonies were then counted, and the colony-forming units per milliliter (CFU/mL) for each sample were recorded. Triplicates of each plate were inoculated and the average number of colony counts (in CFU/mL) was reported.

#### 2.2.2. Enumeration of *Escherichia coli, Salmonella* and *Vibrio*

Diluted samples were inoculated on MacConkey agar (HIMEDIA M081B—India) plates, bismuth sulfite agar (HIMEDIA M027—India) plates and thiosulfate-citrate-bile salts-sucrose (TCBS) agar (HIMEDIA M189—India), for the enumeration of *E. coli*, *Salmonella* and *Vibrio* species, respectively. Triplicates of all the plates were performed and incubated at 37 °C for 24 h. After incubation, colonies were counted, and the average bacterial counts for three different species in terms of CFU/mL were recorded.

#### 2.2.3. Isolation and Preservation of Microorganisms

Single colonies from each type of plate viz. MacConkey agar, bismuth sulfite agar and TCBS agar were streaked on nutrient agar plates. The plates were kept at 37 °C for 24 h to obtain isolated pure colonies. Isolated colonies were preserved in 50% glycerol stock and then stored at −20 °C.

### 2.3. Antibiotic Susceptibility Test (AST)

All the bacterial isolates were tested for antimicrobial susceptibility using five different commonly used antibiotics, ampicillin (HIMEDIA-India), cefotaxime (BD), chloramphenicol (HIMEDIA-India), ciprofloxacin (HIMEDIA-India), and imipenem (BD). Mueller–Hinton agar (HIMEDIA M173) plates were prepared and isolated colonies were streaked on them using sterile swabs. Antibiotic discs were inserted at proper intervals in the plate. Plates were then incubated at 37 °C for 24 h. Test culture zone diameter (in mm) was measured using the antibiotic inhibition chart (Clinical and Laboratory Standards Institute (CLSI) document M100-S23 (M02-A11)).

### 2.4. Statistical Analysis

Statistical analyses were conducted using IBM SPSS (IBM Corporation, Armonk, NY, USA) Statistics for Windows, Version 24. Summary statistics for positive and negative (i.e., safe and unsafe) hygiene behavior of the vendor participants were calculated as frequencies and percentages, and chi-square tests were done for categorical variables. Responses were analyzed to determine the number and percentage of participants that responded to each question, and the locations where vendors reported the highest frequency and percentage for a particular behavior. For bacterial count numbers (continuous variables), means and standard deviations have been reported. Error bars have been described in each figure. Statistical significance was defined as *p* < 0.05. A post hoc power calculation was conducted to estimate the power of the chi-square tests, and to identify the minimum sample size required to achieve a power of 80% at 5% alpha level. Our average effect size was 0.37. Post hoc power analysis was calculated using G*Power 3.1 [30]

## 3. Results

Out of the fifty vendors interviewed, all the vendors were male, and 50% were aged between 30–40 years. Education profile of vendors showed that 44% of the vendors were illiterate.

### 3.1. Hygiene Behavior of the Street Juice Vendors

Safe and unsafe hygiene practices followed by the vendors during the extraction and preparation of juice samples are summarized in Table 1. Overall, safe material handling and hygiene practices among the vendors were mixed. Among the safe practices, the majority of the vendors in DU (North campus) and KN confirmed that they washed hands and utensils with soap and water, whereas more than 60% of vendors showed such positive hygiene behaviors in other locations. However, many vendors demonstrated unsafe practices, such as wiping their hands on their clothing.

Unsafe behavior such as tobacco chewing was found to be highest among the vendors in CC and lowest in RO. Smoking was found to be highest in TN and RO region, while lowest in DU. Vendors from CC demonstrated several negative behaviors, including scalp scratching and wiping their hands on their clothes. Another unsafe behavior practiced by the majority of the participants from CC and KN vendors included storing peeled fruits in open containers, which was low among the vendors from TN and RO. On the other hand, DU street vendors exhibited several positive hygiene behaviors. Hand washing with soap and water, a positive, safe hygiene behavior was found to be highest among the vendors of DU. Additionally, peeling the fruits just before extraction, using closed containers to store their sugar and spice mix (also observed in high frequency among KN vendors), using insect killers to kill mosquitoes, and using waste disposal containers with lids (also observed among TN vendors) were also seen in DU.

The use of municipal supply water or ice prepared from this water, which was often reported to contain pathogenic bacteria [31], was very common among the participating vendors, producing an opportunity of microbial exposure for the customers. This study found that almost 95% of juice vendors used municipal water for washing fruits and preparing juice drinks. Only a small number of DU vendors were found to use packaged mineral water for dilution or ice preparation. Overall, DU vendors had better hygiene practices than the vendors from other study areas. Hygiene behavior data also suggest that vendors’ self-hygiene practices varied greatly across the five markets, reflecting the lack of knowledge about clean and safe working environment among the fruit juice vendors in Delhi street markets.

Our results, however, need to be interpreted with caution, since a post hoc power analysis showed that our chi-square tests were mostly underpowered, with only 5 tests achieving greater than 80% power. We lacked adequate sample size, and required a sample of 88 participants to attain 80% power for this study.

### 3.2. Bacterial Isolates and AMR Characteristics in Juice Samples

**Figure 1:**
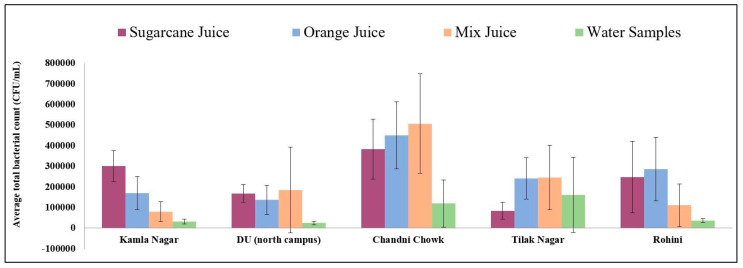
Average total bacterial count (colony forming units (CFU)/mL) in different fruit juices for five most famous markets of Delhi.

The average total bacterial count (TBC) in juice and water samples shown in Figure 1 indicated that TBC was the highest in the samples from CC and lowest in DU. The highest average TBC in water samples was found in TN, and the lowest in DU.

High frequencies of *E. coli*, *Salmonella* and *Klebsiella* spp. were detected in the majority of juice samples (Table 2). A high frequency of *E. coli* spp. was detected in sugarcane juice samples from CC and RO, although the frequency was low in sugarcane juice from TN. In mix juice samples, *E. coli* spp. was found to be highest in CC and lowest in RO and DU. A high frequency of *Vibrio* spp. was detected in orange juice samples from TN and RO. Overall, we observed lower microbial load for *Vibrio* spp. in all juice products when compared with *E. coli* and *Salmonella* spp.

**Figure 2:**
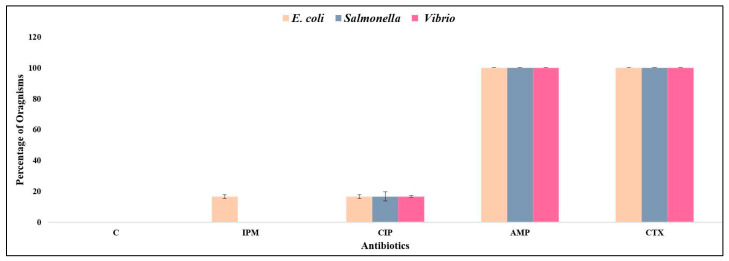
Antimicrobial resistance (AMR) for five different antibiotics (C—chloramphenicol, IPM—imipenem, CIP—ciprofloxacin, AMP—ampicillin, CTX—cefotaxime) in three pathogenic bacteria.

All isolates (i.e., 100%) of *E. coli*, *Salmonella* and *Vibrio* were found to be resistant to ampicillin and cefotaxime, although no resistance was detected for chloramphenicol (Figure 2). Around 16.7% isolates of *E. coli* were found to be resistant to imipenem, whereas 16.7% of all three types of bacterial species were found to be resistant to ciprofloxacin.

## 4. Discussion

Street food vendors sell products to millions of LMIC consumers at a reasonable cost, thus contributing to the economic growth in these countries. However, such healthy input to the economy can be jeopardized by the unsafe hygiene practices of the vendors, as they create opportunities for infectious diseases. In India, the Food Safety Standard Authority of India (FSSAI) in collaboration with the Ministry of Skill Development and Entrepreneurship, launched a “clean street food” education program to train 20,000 roadside vendors about safe food preparation [32]. According to WHO, unsafe practices such as chewing or smoking tobacco do not only affect the health of the vendors, but also put consumers at risk of exposure to toxic chemicals (e.g., second hand tobacco smoke exposure containing polycyclic aromatic hydrocarbons) [33]. *E. coli* and *Salmonella* can survive on finger tips in contaminated hands of the vendors for long time periods [33]. Another key aspect of safe food handling practice is washing hands with water and soap, or even just water, because the dirty hands of the vendors can act as carriers of pathogenic *E. coli*, *Salmonella* or *Vibrio*, and transmit them to consumers [8,34,35]. The results of the current study are in agreement with the findings of the previous studies, and therefore, justify why it is so important to implement educational programs for street food vendors across India and in other developing countries. In our study, it is encouraging to note that more than 60% of vendors from each market were found to wash hands with soap and water. However, several other unsafe behaviors, including frequent itching of the body, wiping of hands on their dirty clothes, and scalp scratching during the preparation of juice samples were also observed, which could potentially induce heavy microbial contamination in the juice products.

The importance of source water and waste disposal are crucial in offering safe juice products to consumers [33,36,37]. Several studies in India have reported the significant role of water quality, as water is used for diluting the raw juices [38,39]. Our study observed that almost 100% of vendors used water from municipal sources, and bought ice from local vendors. The source water samples from CC and TN showed high TBC. Consequently, high frequencies of *E. coli* and *Salmonella* were detected in juice samples in these two market areas. Various other practices, such as the inadequate washing of utensils, the peeling time of fruits and the inappropriate storage of peeled fruits also contribute to the increased probability of microbial contamination. Approximately 40% of vendors peeled fruits at the time of juice preparation, while others preferred to peel fruit in advance, but kept them in open containers, which facilitated its exposure to flies and other pathogen carrying vectors. Improper disposal of waste is another key source of flies and mosquitoes. Moreover, more than 40% of vendors kept waste bins without a lid, and a very high number of vendors did not even have waste bins, and dumped garbage in and around their stall on the roadside.

The variability of the loads of the three microbial species across the study locations is an important finding of the study. *E. coli* load in freshly prepared orange juice samples was much lower than juice prepared a few hours or even a few days ago. This is consistent with findings reported in previous studies showing *E. coli*’s ability to survive around 6–9 days in orange juice [40,41]. High loads of *Salmonella* have been reported in orange juice samples from Mexico and the United States [15,16]. Our study also demonstrated high numbers of *Salmonella* species in both orange and mix juice samples, especially in the CC and TN areas. High *Salmonella* load was correlated with high TBC in source water used for preparation of juice and poor hygiene practices of the vendors at these two locations. On the contrary, no *Salmonella* was found in orange juice as well as mix and sugarcane juice samples from KN. The lower *Salmonella* load was also correlated with the lower TBC of the source water used in this area. A similar low microbial risk pattern was observed in juice and water samples from DU. Apart from orange juice collected at TN and RO and sugarcane juice sold by the vendors of TN, the presence of *Vibrio* contamination in our study areas were negligible. Previous studies also found the similar low *Vibrio* contamination of fruit juices when compared with *E. coli* and *Salmonella* contamination [24,42,43].

The findings in our study regarding AMR characteristics of the three isolated microbial species present further evidence regarding a pressing and emerging public health crisis in LMICs. Overall, we found a high resistance to ampicillin and cefotaxime in *E. coli*, *Salmonella* and *Vibrio*, whereas a low resistance was observed to ciprofloxacin. Resistance to imipenem was observed only in *E. coli* strains, but no resistance was detected against chloramphenicol. These findings differed from another study conducted in Wardha city of Maharashtra, India, which reported AMR to chloramphenicol in *E. coli* and *Salmonella* strains, although that study reported no resistance to ciprofloxacin [44]. In another study conducted in Dhaka, Bangladesh, that has similar sociocultural and environmental characteristics to urban Delhi, ampicillin- and chloramphenicol-resistant strains of *E. coli*, *Salmonella* and *Vibrio* were identified in orange juice samples [45,46].

Our data on AMR to ampicillin and cefotaxime for *E. coli*, *Salmonella* and *Vibrio* are clinically important, since these antibiotics are on WHO’s list of essential medicine [47]. Both antibiotics are used to treat common infections, such as urinary tract and respiratory infections, meningitis, and neonatal sepsis [48,49]. Ampicillin is known to be widely used to treat *Shigella* and *Salmonella* infections, which are prominent in developing nations, including India [50]. On the other hand, cefotaxime is an extended-spectrum cephalosporin that has a wide range of antibiotic therapy and imparts less adverse side effects [49]. Although WHO has recommended that cefotaxime might not be the right choice for the treatment of shigellosis in Asia and Africa, it is widely used to treat meningitis in India [51]. Overall, ampicillin and cefotaxime are crucial and standard drugs of choice to treat clinically significant infectious organisms such as *E. coli*, *Staphylococcus aureus*, *Salmonella typhi*, *Shigella* spp., *Streptococcus pneumoniae* and *H. influenzae*.

There are several potential limitations in our study. This was an exploratory study, and therefore no absolute conclusive statement can be drawn from the results. The major goal of the study was to generate pilot data regarding the antibiotic resistant bacteria present in fruit juice products prepared and sold by the street food vendors in a crowded city in India (i.e., Delhi). Our sample size was also restricted, since we lacked adequate funding. A post hoc power analysis revealed that our chi-square tests were mostly underpowered and we had insufficient sample size. In order to achieve 80% power in this study, we needed a total sample size of 88 (around 18 subjects per study area). Nevertheless, our study results indicate the presence of bacteria resistant to several clinically significant antibiotics in the juice samples, which is crucial. We also only interviewed juice vendors from crowded street markets, who often work from visibly unhygienic areas. We were unable to compare the microbial quality of the juices from these markets with those from more upscale markets, as it was beyond the scope of this project. Therefore, the study results may not be generalizable to the rest of India and other LMICs. This study also relied on self-reported data, which may lead to the overestimation of positive hygiene behavior practices, due to social desirability. However, fruit juice preparation and handling practices were also directly observed and recorded to minimize self-reporting bias. Additionally, only classical microbiological analysis measures were used in this study, which may lead to a lack of robust data. The lack of a comparison group is another limitation in this study. However, our aim was to obtain a snapshot of the current hygiene practices of the juice vendors and microbiological quality of the fruit juices. In future, we plan to expand the study with a larger sample size, including a comparison group, with a collection of samples over different seasons of the year. We plan to use high throughput analytic methods to elucidate the flux of antibiotic resistant bacteria in future large-scale studies.

## 5. Conclusions

The microbiological quality of fruit juices served by street vendors in Delhi was not within the acceptable limits, as reflected in our study. Furthermore, water quality used for the preparation of juice products was compromised in most of the study locations. Bacterial strains isolated from juice samples were resistant to some common and essential antibiotics, indicating a pressing public health concern. Additionally, the street food practices and personal hygiene of vendors across five study locations in Delhi were not even close to the national standards. Findings of the study emphasize the need of stringent monitoring and regulations regarding street fruit juice preparation by the Ministry of Health in urban India, since millions of consumers are heavily relying on these products. Moreover, we have observed antibiotic resistance in the strains of *E. coli*, *Salmonella* and *Vibrio* isolated from fruit juice samples against two clinically crucial antibiotics, ampicillin, and cefotaxime. Based on our findings, future studies should focus on developing awareness programs to educate and improve hygiene and food handling behaviors practiced by fruit juice vendors. There is an urgent need for public health agencies and practitioners working on food safety to develop strategies and intervention plans to provide free training to the street food vendors on food safety, sanitation standards and hygiene behavior. Policymakers should frame policies to ensure that basic amenities such as clean water supply and proper disposal of garbage are available, so that the street fruit juices only offer rich sources of vitamins, minerals and antioxidants, and do not serve as reservoirs of pathogenic and antibiotic resistant microorganisms.

## Figures and Tables

**Table 1:** Hygiene behavior among the fruit juice vendors from five study areas in urban Delhi.

Hygiene Behavior	Locations Showing the Highest % of Behavior	Highest Frequency of Response at a Location/Total Response	Combined Response of all Five Locations (*n* = 50) *n* (%)	Comparison between all Locations *p*-Values
**Positive Behavior**				
Hand washing with water	Kamla Nagar and Chandni Chowk	3/10	10 (20)	0.44
Hand washing with soap and water	University of Delhi (DU) (North Campus)	10/10	40 (80)	0.44
Utensil washing with water	Chandni Chowk	7/10	19 (38)	0.13
Utensil washing with soap and water	Kamla Nagar and DU (North Campus)	8/10	31 (62)	0.12
Storage of peeled fruits in closed container	Kamla Nagar, Tilak Nagar and Rohini	2/10	10 (20)	0.99
Peeling of fruit just before juice extraction	DU (North Campus)	7/10	21 (42)	0.26
Keep grinded sugar in closed container	Kamla Nagar and DU (North Campus)	8/10	29 (58)	0.01 *
Uses packaged drinking water	DU (North Campus)	1/10	1 (2)	NA
Uses mosquito spray	DU (North Campus) and Tilak Nagar	6/10	22 (44)	0.13
Uses waste bin with lid	DU (North Campus) and Tilak Nagar	6/10	22 (44)	0.14
**Negative Behavior**				
Uses already peeled fruits	Kamla Nagar	8/10	29 (58)	0.26
Keep peeled fruits in open container	Kamla Nagar	7/10	24 (48)	0.36
Keep grinded sugar in open container	Chandni Chowk	9/10	21 (42)	0.01 *
Uses municipal water	Kamla Nagar, Tilak Nagar, Rohini and Chandni Chowk	10/10	49 (98)	0.39
Uses ice bought from local ice vendor	Chandni Chowk, Tilak Nagar and Rohini	9/10	40 (80)	0.11
Uses ice from own source of water	Kamla Nagar	5/10	10 (20)	0.12
Do not use mosquito spray	Chandni Chowk	9/10	28 (56)	0.14
Do not use bin	Chandni Chowk	7/10	17 (34)	0.02 *
Uses bin without lid	Kamla Nagar	4/10	11 (22)	0.41
Tobacco chewing	Chandni Chowk	10/10	32 (64)	0.03 *
Scalp scratching	Chandni Chowk	3/10	11 (22)	0.97
Itching on body parts	Kamla Nagar and Rohini	1/10	2 (4)	NA
Wiping hands with clothing	Chandni Chowk	8/10	21 (42)	0.05

* Differences between locations were statistically significant (*p* < 0.05).

**Table 2:** Average total count (CFU/mL) of *E. coli, Salmonella and Vibrio* in fruit juices in five different locations.

	Sugarcane Juice			Orange Juice			Mix Juice		
	*E. coli* (CFU/mL)	*Salmonella* (CFU/mL)	*Vibrio* (CFU/mL)	*E. coli* (CFU/mL)	*Salmonella* (CFU/mL)	*Vibrio* (CFU/mL)	*E. coli* (CFU/mL)	*Salmonella* (CFU/mL)	*Vibrio* (CFU/mL)
Kamla Nagar	3.5 × 10^4^ (±39,393.5)	5.5 × 10^4^ (±33,842.7)	6.3 × 10^2^ (±664.2)	1.3 × 10^4^ (±12,834.7)	0	4.4 × 10^3^ (±3413.6)	5.7 × 10^4^ (±61,675.7)	4.8 × 10^4^ (±66,468.0)	0
DU (North campus)	9.7 × 10^4^ (±58,488.6)	6.3 × 10^4^ (±8838.8)	8.2 × 10^2^ (±56.5)	1.6 × 10^4^ (±2886.7)	1.1 × 10^4^ (±12,288.2)	1 × 10^3^ (±1979.8)	1.3 × 10^4^ (±1044.03)	7.3 × 10^3^ (±318.19)	4.3 × 10^3^ (±296.9)
Chandni Chowk	2 × 10^5^ (±62,010.7)	9.3 × 10^4^ (±49,369.3)	4.8 × 10^3^ (±2709.8)	1.7 × 10^5^ (±127,719)	1.8 × 10^5^ (±111,519)	1.5 × 10^3^ (±494.9)	1.7 × 10^5^ (±158,104)	2.2 × 10^5^ (±127,899)	1.4 × 10^3^ (±253.8)
Tilak Nagar	4 × 10^4^ (±2309.4)	1.6 × 10^5^ (±133,180)	1.1 × 10^4^ (±15,044.3)	3.7 × 10^4^ (±1778.5)	7.5 × 10^4^ (±84,902.3)	7.7 × 10^4^ (±87,726.6)	1.7 × 10^5^ (±150,414)	1.1 × 10^5^ (±75,438.7)	4.5 × 10^4^ (±36,693.7)
Rohini	1.8 × 10^5^ (±216,569)	8.4 × 10^4^ (±118,392)	3.2 × 10^3^ (±3364.5)	6.1 × 10^4^ (±32,896.1)	9.6 × 10^4^ (±47,619.2)	3 × 10^4^ (±2474.8)	2.5 × 10^4^ (±20,297.7)	2.6 × 10^4^ (±4596.1)	0
